# An Improved Recurrent Neural Network for Complex-Valued Systems of Linear Equation and Its Application to Robotic Motion Tracking

**DOI:** 10.3389/fnbot.2017.00045

**Published:** 2017-09-01

**Authors:** Lei Ding, Lin Xiao, Bolin Liao, Rongbo Lu, Hua Peng

**Affiliations:** ^1^College of Information Science and Engineering, Jishou University, Jishou, China

**Keywords:** complex-valued systems of linear equation, recurrent neural network, finite-time convergence, robot, gradient neural network, motion tracking

## Abstract

To obtain the online solution of complex-valued systems of linear equation in complex domain with higher precision and higher convergence rate, a new neural network based on Zhang neural network (ZNN) is investigated in this paper. First, this new neural network for complex-valued systems of linear equation in complex domain is proposed and theoretically proved to be convergent within finite time. Then, the illustrative results show that the new neural network model has the higher precision and the higher convergence rate, as compared with the gradient neural network (GNN) model and the ZNN model. Finally, the application for controlling the robot using the proposed method for the complex-valued systems of linear equation is realized, and the simulation results verify the effectiveness and superiorness of the new neural network for the complex-valued systems of linear equation.

## 1 Introduction

Today, the complex-valued systems of linear equation has been applied into many fields (Duran-Diaz et al., 2011; Guo et al., 2011; Subramanian et al., 2014; Hezari et al., 2016; Zhang et al., 2016; Xiao et al., 2017a). In mathematics, the complex-valued systems of linear equations can be written as

(1) $$\mathrm{Az}\left(t\right)=b\in C^{n},$$

where $A\in C^{n\times n}$ and $b\in C^{n}$ are the complex-valued coefficients, and $z\left(t\right)\in C^{n}$ is a complex-valued vector to be computed. Xiao et al. (2015) proposed a fully complex-valued gradient neural network (GNN) to solve such a complex-valued systems of linear equation. However, the corresponding error norm usually converges to the theoretical solution after very long time. So to increase the convergence rate, a kind of neural network called Zhang neural network (ZNN) is proposed to make the lagging error converge to 0 exponentially (Zhang and Ge, 2005; Zhang et al., 2009). However, in Xiao (2016) and Xiao et al. (2017b), Xiao pointed that the original ZNN model cannot converge to 0 within finite time, and its real-time calculation capability may be limited (Marco et al., 2006; Li et al., 2013; Li and Li, 2014; Xiao, 2015). So, Xiao (2016) presented a new design formula, which can converge to 0 within finite time for the time-varying matrix inversion.

Considering that a complex variable can be written as the combination of its real and imaginary parts, we have *A* = *A*_re_ + *jA*_im_, *b* = *b*_re_ + *jb*_im_, and *z*(*t*) = *z*_re_(*t*) + *z*_im_(*t*), where the symbol $j=\sqrt{-1}$ means an imaginary unit. Therefore, the equation (1) can be presented as

(2) $$\left[A_{\text{re}}+{\mathrm{jA}}_{\text{im}}\right]\left[z_{\text{re}}\left(t\right)+{\mathrm{jz}}_{\text{im}}\left(t\right)\right]=b_{\text{re}}+{\mathrm{jb}}_{\text{im}}\in C^{n},$$

where $A_{\text{re}}\in R^{n\times n},A_{\text{ im}}\in R^{n\times n}$, $z_{\text{re}}\in R^{n},z_{\text{ im}}\in R^{n}$, $b_{\text{re}}\in R^{n}$, and $b_{\text{im}}\in R^{n}$. According to the complex formula, the real (or imaginary) part of the left-side and right-side of equation is equal (Zhang et al., 2016). Then we have

(3) $$\left\{\begin{array}{c} A_{\text{re}}z_{\text{re}}\left(t\right)-A_{\text{im}}z_{\text{im}}\left(t\right)=b_{\text{re}}\in R^{n},\\ A_{\text{im}}z_{\text{re}}\left(t\right)+A_{\text{re}}z_{\text{im}}\left(t\right)=b_{\text{im}}\in R^{n}. \end{array}\right.$$

Thus, we can express the equation (3) in a compact matrix form as:

(4) $$\left[\begin{array}{cc} A_{\text{re}} & -A_{\text{im}}\\ A_{\text{im}} & A_{\text{re}} \end{array}\right]\left[\begin{array}{c} z_{\text{re}}\left(t\right)\\ z_{\text{im}}\left(t\right) \end{array}\right]=\left[\begin{array}{c} b_{\text{re}}\\ b_{\text{im}} \end{array}\right]\in R^{2n}.$$

We can write the equation (4) as

(5) $$\mathrm{Cx}\left(t\right)=e\in R^{2n},$$

where $C=\left[\begin{array}{cc} A_{\text{re}} & -A_{\text{im}}\\ A_{\text{im}} & A_{\text{re}} \end{array}\right]$, $x\left(t\right)=\left[\begin{array}{c} z_{\text{re}}\left(t\right)\\ z_{\text{im}}\left(t\right) \end{array}\right]$, and $e=\left[\begin{array}{c} b_{\text{re}}\\ b_{\text{im}} \end{array}\right]$. Now the complex-valued system of linear equation can be computed in real domain. In this situation, most methods for solving real-valued system of linear equation can be used to solve the complex-valued system of linear equation (Zhang and Ge, 2005; Zhang et al., 2009; Guo et al., 2011). For example, a gradient neural network (GNN) can be designed to solve such a real-valued system of linear equation. The GNN model can be directly presented as follows (Xiao et al., 2015):

(6) $$\dot{x}\left(t\right)=-\gamma C^{\text{T}}\left(\mathrm{Cx}\left(t\right)-e\right),$$

where design parameter γ > 0 is employed to adjust the convergence rate of the GNN model. Zhang et al. (Zhang et al., 2016) used the recurrent neural network to solve the complex-valued quadratic programming problems. Hezari et al. (2016) solved a class of complex symmetric system of linear equations using an iterative method. However, the above mentioned neural networks cannot converge to the desired solution within finite time. Considering that the complex-valued system of linear equation can be transformed into the real-valued system of linear equation, a new neural network can be derived from the new design formula proposed by Xiao for solving the complex-valued system of linear equation (Xiao et al., 2015). In addition, the new neural network possesses a finite-time convergence property.

In recent years, the research on robot has become a hot spot (Khan et al., 2016a,b; Zanchettin et al., 2016; Guo et al., 2017), and the neural network has been successfully applied into the robot domain (He et al., 2016; Jin and Li, 2016; Woodford et al., 2016; Jin et al., 2017; Xiao, 2017). However, the application of the new design method for the complex-valued system of linear equation in robot domain has not been reported. So this is the first time to propose a new neural network, which can convergence within finite-time for solving the complex-valued system of linear equation and its application to robot domain.

The rest of this paper is organized into four sections. Section 2 proposes a finite-time recurrent neural network (FTRNN) to deal with the complex-valued system of linear equation, and its convergence analysis is given in detail. Section 3 gives the computer-simulation results to substantiate the theoretical analysis and the superiority. Section 4 gives the results of the application for controlling the robotic motion planning. Finally, the conclusions are presented in Section 5. Before ending this section, the main contributions of the current work are presented as follows.

- The research object focuses on a complex-valued system of linear equation in complex domain, which is quite different from the previously investigated real-valued system of linear equation in real domain.
- A new finite-time recurrent neural network is proposed and investigated for solving complex-valued systems of linear equation in complex domain. In addition, it is theoretically proved to be convergent within finite time.
- Theoretical analyses and simulative results are presented to show the effectiveness of the proposed finite-time recurrent neural network. In addition, a five-link planar manipulator is used to validate the applicability of the finite-time recurrent neural network.

## 2 Finite-Time Recurrent Neural Network

Considering that the complex-valued system of linear equation can be computed in real domain, the error function *E*(*t*) of traditional ZNN can be presented as

(7) $$E\left(t\right)=\mathrm{Cx}\left(t\right)-e\in R^{2n}.$$

Then, according to the design formula $\dot{E}\left(t\right)=-\gamma \text{Φ}\left(E\left(t\right)\right)$, the original ZNN model can be presented as

(8) $$C\dot{x}\left(t\right)=-\gamma \text{Φ}\left(\mathrm{Cx}\left(t\right)-e\right),$$

where Φ(·) means an activation function array, and γ > 0 is used to adjust the convergence rate. In this paper, the new design formula in Xiao (2016) for *E*(*t*) can be directly employed and written as follows:

(9) $$\frac{\text{d}E\left(t\right)}{\text{d}t}=-\gamma \text{Φ}\left(\rho_{1}E\left(t\right)+\rho_{2}E^{j∕f}\left(t\right)\right),$$

where the parameters *ρ*_1_ and *ρ*_2_ satisfy *ρ*_1_ > 0, *ρ*_2_ > 0, and *f* and *j* mean the positive odd integer and satisfy *f*  > *j*. Then we have

(10) $$C\dot{x}\left(t\right)=-\gamma \text{Φ}\left(\rho_{1}\left(\mathrm{Cx}\left(t\right)-e\right)+\rho_{2}{\left(\mathrm{Cx}\left(t\right)-e\right)}^{j∕f}\left(t\right)\right).$$

To simplify the formula, Φ(·) uses the linear activation function. Then we have

(11) $$\frac{\text{d}E\left(t\right)}{\text{d}t}=-\gamma \left(\rho_{1}E\left(t\right)+\rho_{2}E^{j∕f}\left(t\right)\right),$$

and

(12) $$C\dot{x}\left(t\right)=-\gamma \left(\rho_{1}\left(\mathrm{Cx}\left(t\right)-e\right)+\rho_{2}{\left(\mathrm{Cx}\left(t\right)-e\right)}^{j∕f}\right),$$

which is called the finite-time recurrent neural network (FTRNN) model to online deal with the complex-valued system of linear equation. In addition, for design formula (11) and FTRNN model (12), we have the following two theorems to ensure their finite-time convergence properties.

**Theorem 1.** *The error function E*(*t*) *of design formula (11) converges to zero within finite-time t_u_ regardless of its randomly generated initial error E*(0)*:*

$$t_{u}=\frac{f}{\gamma \rho_{1}\left(f-j\right)}\ln\frac{\rho_{1}h_{M}{\left(0\right)}^{\left(f-j\right)∕f}+\rho_{2}}{\rho_{2}},$$

*where* *h_M_*(0) *means the maximum element of the matrix E*(0).

Proof. For design formula (11), we have

(13) $$\frac{\text{d}E\left(t\right)}{\text{d}t}=-\left(\gamma \rho_{1}E\left(t\right)+\gamma \rho_{2}E^{j∕f}\left(t\right)\right).$$

To deal with the dynamic response of the equation (13), the above differential equation can be rewritten as below:

(14) $$E^{-j∕f}\left(t\right)◇\frac{\text{d}E\left(t\right)}{\text{d}t}+\gamma \rho_{1}E^{\left(f-j\right)∕f}\left(t\right)=-\gamma \rho_{2},$$

where the matrix-multiplication operator ◇ means the Hadamard product and can be written as

$$W◇S=\left[\begin{array}{cccc} W_{11}S_{11}, & W_{12}S_{12}, & \cdots , & W_{1n}S_{1n}\\ R_{21}S_{21}, & W_{21}S_{21}, & \cdots , & W_{2n}S_{2n}\\ \vdots & \vdots & \ddots & \vdots \\ W_{m1}S_{m1}, & W_{m2}S_{m2}, & \cdots , & W_{\mathrm{mn}}S_{\mathrm{mn}}, \end{array}\right]\in R^{m\times n}.$$

Now let us define *Y* (*t*) = *E*^(^*^f^*^–^*^j^*^)/^*^f^*(*t*). Then, we have

$$\frac{\text{d}Y\left(t\right)}{\text{d}t}=\frac{f-j}{f}E^{-j∕f}\left(t\right)◇\frac{\text{d}E\left(t\right)}{\text{d}t}.$$

Thus, the differential equation (14) can be equivalent to the following first order differential equation:

(15) $$\frac{\text{d}Y\left(t\right)}{\text{d}t}+\frac{f-j}{f}\gamma \rho_{1}Y\left(t\right)=-\frac{f-j}{f}\gamma \rho_{2}I.$$

This is a typical first order differential equation, and we have

(16) $$Y\left(t\right)=\left(\frac{\rho_{2}}{\rho_{1}}I+Z\left(0\right)\right)\exp\left(-\frac{f-j}{f}\gamma \rho_{1}t\right)-\frac{\rho_{2}}{\rho_{1}}I.$$

So we have

(17) $$E^{\left(f-j\right)∕f}\left(t\right)=\left(\frac{\rho_{2}}{\rho_{1}}I+E^{\left(f-j\right)∕f}\left(0\right)\right)\exp\left(-\frac{f-j}{f}\gamma \rho_{1}t\right)-\frac{\rho_{2}}{\rho_{1}}I,$$

and

(18) $$E\left(t\right)={\left[\left(\frac{\rho_{2}}{\rho_{1}}I+E^{\left(f-j\right)∕f}\left(0\right)\right)\exp\left(-\frac{f-j}{f}\gamma \rho_{1}t\right)-\frac{\rho_{2}}{\rho_{1}}\right]}^{f∕\left(f-j\right)}.$$

From the equation (18), we can find the error matrix *E*(*t*) will converge to 0 in *t_u_*, and

(19) $$\left(\frac{\rho_{2}}{\rho_{1}}I+E^{\left(f-j\right)∕f}\left(0\right)\right)\exp\left(-\frac{f-j}{f}\gamma \rho_{1}t_{u}\right)-\frac{\rho_{2}}{\rho_{1}}I=0.$$

Considering each element of the matrix *E*(*t*) has the same identical dynamics, we have

(20) $$t_{\mathrm{ik}}=\frac{f}{\gamma \rho_{1}\left(f-j\right)}\ln\frac{\rho_{1}h_{\mathrm{ik}}^{\left(f-j\right)∕f}\left(0\right)+\rho_{2}}{\rho_{1}},$$

where *h_ik_* means the *ik*th element of the matrix *E*(0), and *t_ik_* means the *ik*th finite-time convergence upper bound of the matrix *E*(*t*). Let *h_M_*(0) = max(*h_ik_*). Then for any *ik*th element of the matrix *E*(*t*), we have the maximum convergence time:

$$t_{u}=\frac{f}{\gamma \rho_{1}\left(f-j\right)}\ln\frac{\rho_{1}h_{M}{\left(0\right)}^{\left(f-j\right)∕f}+\rho_{2}}{\rho_{2}}.$$

According to the above analysis, we can draw a conclusion that the error matrix *E*(*t*) will converge to 0 within the finite time *t_u_* regardless of its initial value *E*(0). Now the proof is completed. □

**Theorem 2.** *The state matrix X*(*t*) *of FTRNN model (12) will converge to the theoretical solution of (5) in finite time t_u_ regardless of its randomly generated initial state x*(0), *and*

$$t_{u}\in \left\{\frac{f}{\gamma \rho_{1}\left(f-j\right)}\ln\frac{\rho_{1}h_{L}{\left(0\right)}^{\left(f-j\right)∕f}+\rho_{2}}{\rho_{2}},\right.\left.\frac{f}{\gamma \rho_{1}\left(f-j\right)}\ln\frac{\rho_{1}h_{M}{\left(0\right)}^{\left(f-j\right)∕f}+\rho_{2}}{\rho_{2}}\right\},$$

*where* *h_M_*(0) *and* *h_L_*(0) *mean the largest and the smallest elements of the matrix E*(0), *respectively*.

Proof. Let *x*_(_*_FT_*_)_(*t*) represent the solution of the FTRNN model (12), *x*_(_*_org_*_)_(*t*) represent the theoretical solution of the equation (5), and $\tilde{x}\left(t\right)$ represent the difference between *x*_(_*_FT_*_)_(*t*) and *x*_(_*_org_*_)_(*t*). Then, we can obtain

(21) $$\tilde{x}(t)=x_{\left(\mathrm{FT}\right)}(t)-x_{\left(\mathrm{org}\right)}(t)\in R^{2n\times 2n}.$$

The equation (21) can be written as

(22) $$x_{\left(\mathrm{FT}\right)}(t)=\tilde{x}(t)+x_{\left(\mathrm{org}\right)}(t)\in R^{2n\times 2n}.$$

Substitutes the above equation into FTRNN model (12), we have

(23) $$C(\dot{\tilde{x}}(t)+{\dot{x}}_{\left(\mathrm{org}\right)}(t))=-\gamma \left(\rho_{1}(C(\tilde{x}(t)+x_{\left(\mathrm{org}\right)}(t))-e)+\rho_{2}{\left(C(\tilde{x}(t)+x_{\left(\mathrm{org}\right)}(t))-e\right)}^{j/f}\right).$$

Considering *Cx*_(_*_org_*_)_(*t*) − *e* = 0 and $C{\dot{x}}_{\left(\mathrm{org}\right)}\left(t\right)=0$, the above equation can be written as

$$C\dot{\tilde{x}}\left(t\right)=-\gamma \left(\rho_{1}\left(C\tilde{x}\left(t\right)-e\right)+\rho_{2}{\left(C\tilde{x}\left(t\right)-e\right)}^{j∕f}\right).$$

Furthermore, considering $E\left(t\right)=C\left(\tilde{x}\left(t\right)+x_{\left(\mathrm{org}\right)}\left(t\right)\right)-e$, *Cx*_(_*_org_*_)_(*t*) − *e* = 0, and $E\left(t\right)=C\tilde{x}\left(t\right)$, the above differential equation can be written as

$$\frac{\text{d}E\left(t\right)}{\text{d}t}=-\gamma \left(\rho_{1}\left(E\left(t\right)-e\right)+\rho_{2}{\left(E\left(t\right)-e\right)}^{j∕f}\right).$$

Let $\tilde{E}\left(t\right)=E\left(t\right)-e$, then we have

(24) $$\frac{\text{d}\tilde{E}\left(t\right)}{\text{d}t}=-\gamma \left(\rho_{1}\tilde{E}\left(t\right)+\rho_{2}{\tilde{E}}^{j∕f}\left(t\right)\right).$$

So according to the equation (20), we have

(25) $${\tilde{t}}_{\mathrm{ik}}=\frac{f}{\gamma \rho_{1}\left(f-j\right)}\ln\frac{\rho_{1}{\overset{˜}{h}}_{\mathrm{ik}}^{\left(f-j\right)∕f}\left(0\right)+\rho_{2}}{\rho_{1}},$$

where ${\tilde{t}}_{\mathrm{ik}}$ means the time upper of *ik*th solution of the matrix $\tilde{E}\left(t\right)$, and ${\tilde{h}}_{\mathrm{ik}}$ means the *ik*th initial error value of the matrix $\tilde{E}\left(0\right)$.

Let us define ${\tilde{h}}_{M}=\text{max}\left({\tilde{h}}_{\mathrm{ik}}\left(0\right)\right)$, and ${\tilde{h}}_{L}=\text{min}\left({\tilde{h}}_{\mathrm{ik}}\left(0\right)\right)$ with *i, k* = 1, 2, … *n*. Then for all possible *i* and *k*, we have

$$\frac{f}{\gamma \rho_{1}\left(f-j\right)}\ln\frac{\rho_{1}{\overset{˜}{h}}_{L}^{\left(f-j\right)∕f}\left(0\right)+\rho_{2}}{\rho_{1}}⩽{\tilde{t}}_{\mathrm{ik}}\left(t\right)⩽\frac{f}{\gamma \rho_{1}\left(f-j\right)}\ln\frac{\rho_{1}{\overset{˜}{h}}_{M}^{\left(f-j\right)∕f}\left(0\right)+\rho_{2}}{\rho_{1}}.$$

The above equation shows that the state matrix $\tilde{x}\left(t\right)=x_{\left(\mathrm{FT}\right)}\left(t\right)-x_{\text{(}\mathrm{org}\text{)}}\left(t\right)$ will converges to 0 within finite time regardless of its initial error value. In another word, the matrix *x*_(*FT*)_(*t*) for the FTRNN model (12) will converge to the theoretical solution *x*_(_*_org_*_)_(*t*) for the theoretical model (5) within finite time regardless of its randomly generated initial state *x*(0). Now the proof is completed. □

## 3 Computer Simulation

In this section, a digital example will be carried out to show the superiority of FTRNN model (12) to GNN model (6) and ZNN model (8). We can choose the design parameters *f* and *j*, which satisfy *f*  > *j*. For example, we choose *f*  = 5 and *j* = 1 in this paper. In addition to this, to substantiate the superiority of FTRNN model (12), we choose the same complex-valued matrix *A* and *b* as these of the paper (Xiao et al., 2015). Then we have

$$A=\left[\begin{array}{cccc} -0.7597+0.6503j & \;\;\;-0.8391-0.5440j & \;\;\;0.2837-0.9589j & \;\;\;1\\ 0.7597+0.6503j & \;\;\;-0.8391+0.5440j & \;\;\;-0.2837-0.9589j & \;\;\;1\\ 0.7597-0.6503j & \;\;\;-0.8391-0.5440j & \;\;\;-0.2837+0.9589j & \;\;\;1\\ 0-1.0000j & \;\;\;-1.0000 & \;\;\;0+1.0000j & \;\;\;1 \end{array}\right].$$

So we have

$$A_{\text{re}}=\;\;\left[\begin{array}{cccc} -0.7597 & -0.8391 & 0.2837 & 1\\ 0.7597 & -0.8391 & -0.2837 & 1\\ 0.7597 & -0.8391 & -0.2837 & 1\\ 0 & -1.0000 & 0 & 1 \end{array}\right],$$

and

$$A_{\text{im}}=\;\;\left[\begin{array}{cccc} 0.6503 & -0.5440 & -0.9589 & 0\\ 0.6503 & 0.5440 & -0.9589 & 0\\ -0.6503 & -0.5440 & 0.9589 & 0\\ -1.0000 & 0 & 1.0000 & 0 \end{array}\right].$$

Now the randomly generated vector *b* = [1.0000, 0.2837 + 0.9589*j*, 0.2837 − 0.9589*j*, 0]^T^ in Xiao et al. (2015) is employed in this paper. The theoretical solution of the complex-valued linear equation system can be written as *z*_(_*_org_*_)_ = [−0.4683−0.2545*j*, 1.2425 + 0.3239*j*, −0.6126 + 0.0112*j*, 1.5082 + 0.4683*j*]. Then according to the equation (5), we have

$$C=\;\;\left[\begin{array}{cccccccc} -0.7597 & \;\;\;-0.8391 & \;\;\;0.2837 & \;\;\;1 & \;\;\;-0.6503 & \;\;\;0.5440 & \;\;\;0.9589 & \;\;\;0\\ 0.7597 & \;\;\;-0.8391 & \;\;\;-0.2837 & \;\;\;1 & \;\;\;-0.6503 & \;\;\;-0.5440 & \;\;\;0.9589 & \;\;\;0\\ 0.7597 & \;\;\;-0.8391 & \;\;\;-0.2837 & \;\;\;1 & \;\;\;0.6503 & \;\;\;0.5440 & \;\;\;-0.9589 & \;\;\;0\\ 0 & \;\;\;-1.0000 & \;\;\;0 & \;\;\;1 & \;\;\;1.0000 & \;\;\;0 & \;\;\;-1.0000 & \;\;\;0\\ 0.6503 & \;\;\;-0.5440 & \;\;\;-0.9589 & \;\;\;0 & \;\;\;-0.7597 & \;\;\;-0.8391 & \;\;\;0.2837 & \;\;\;1\\ 0.6503 & \;\;\;0.5440 & \;\;\;-0.9589 & \;\;\;0 & \;\;\;0.7597 & \;\;\;-0.8391 & \;\;\;-0.2837 & \;\;\;1\\ -0.6503 & \;\;\;-0.5440 & \;\;\;0.9589 & \;\;\;0 & \;\;\;0.7597 & \;\;\;-0.8391 & \;\;\;-0.2837 & \;\;\;1\\ -1.0000 & \;\;\;0 & \;\;\;1.0000 & \;\;\;0 & \;\;\;0 & \;\;\;-1.0000 & \;\;\;0 & \;\;\;1 \end{array}\right],$$

and *e* = [1.0000, 0.2837, 0.2837, 0, 0, 0.9589, −0.9589, 0]^T^. So the theoretical solution of the complex-valued linear equation system can be rewritten as *x*_(_*_org_*_)_ = [−0.4683, 1.2425, −0.6126, 1.5082, −0.2545, 0.3239, 0.0112, 0.4683]^T^.

First, a zero initial complex-valued state $z\left(0\right)\in C^{4}$ is generated, which can be transformed into the real-valued state $x\left(0\right)\in R^{8}$ in real domain. To help facilitate the contrast, we choose the design parameter γ = 5 and γ = 500, respectively.

**Figure 1:**
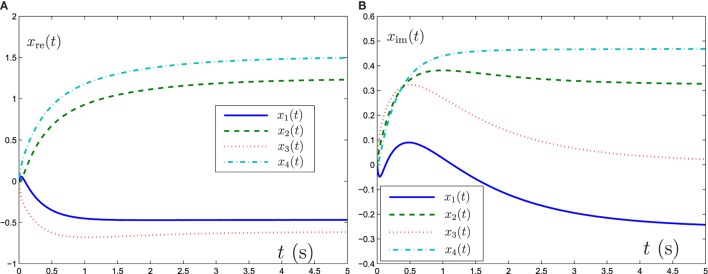
Output trajectories of neural states *x*(*t*) synthesized by GNN model (6) with γ = 5. **(A)** Element of real part of *x*(*t*), **(B)** element of imaginary part of *x*(*t*).

**Figure 2:**
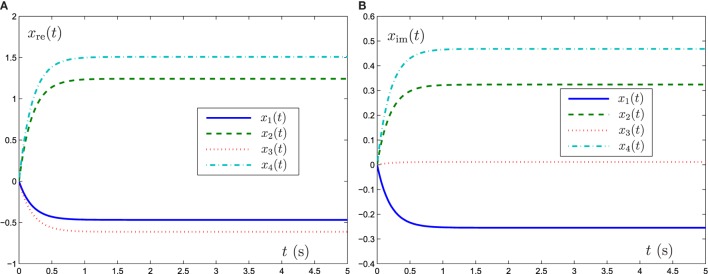
Output trajectories of neural states *x*(*t*) synthesized by ZNN model (8) with γ = 5. **(A)** Element of real part of *x*(*t*), **(B)** element of imaginary part of *x*(*t*).

**Figure 3:**
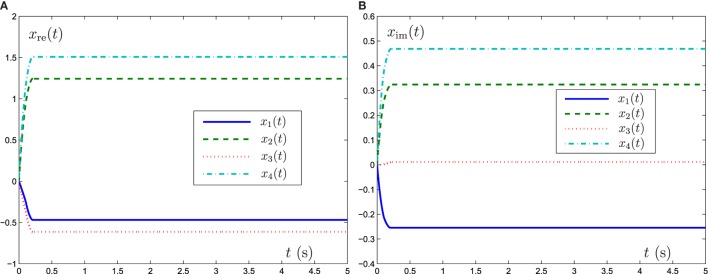
Output trajectories of neural states *x*(*t*) synthesized by FTRNN model (12) with γ = 5. **(A)** Element of real part of *x*(*t*), **(B)** element of imaginary part of *x*(*t*).

Now GNN model (6), ZNN model (8), and FTRNN model (12) are applied to solve this complex-valued systems of linear equation problem. The output trajectories of the corresponding neural-state solutions are displayed in Figures 1–3. As seen from such three figures, we can conclude that the output trajectories of the neural-state solutions can converge to the theoretical solutions, but the convergence rates are different. By comparison, we can easily find that FTRNN model (12) has a fastest convergence property.

**Figure 4:**
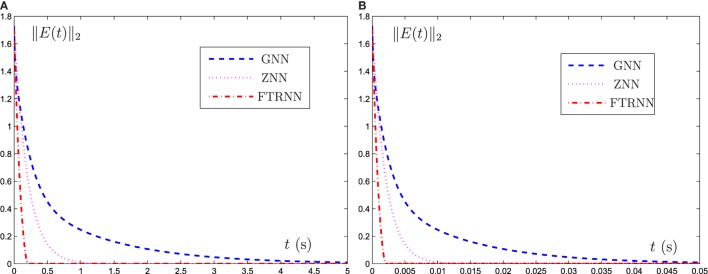
Output trajectories of residual functions ||*E*(*t*)||_2_ synthesized by different neural-network models with **(A)** γ = 5 and **(B)** γ = 500.

To directly show the solution process of such three neural-network models, the evolution of the corresponding residual errors, measured by the norm ||*E*(*t*)||_2_, is plotted in Figure 4 under the conditions of γ = 5 and γ = 500. From Figure 4A, the results are consistent with those of Figures 1–3. In addition, from Figure 4B, the convergence speeds of GNN model (6), ZNN model (8), and FTRNN model (12) can be improved as the value of γ increases.

Now we can draw a conclusion that, as compared with GNN model (6) and ZNN model (8), FTRNN model (12) has the most superiority for solving the complex-valued system of linear equation problem.

## 4 Application to Robotic Motion Tracking

In this section, a five-link planar manipulator is used to validate the applicability of the finite-time recurrent neural network (FTRNN) (Zhang et al., 2011). It is well known that the kinematics equations of the five-link planar manipulator at the position level and at the velocity level are, respectively, written as follows (Xiao and Zhang, 2013, 2014a,b, 2016; Xiao et al., 2017c):

(26) r(t)=f(θ(t))

(27) $$\dot{r}\left(t\right)=J\left(\theta \right)\dot{\theta }\left(t\right)$$

where *θ* denotes the angle vector of the five-link planar manipulator, *r*(*t*) denotes the end-effector position vector, *f* (·) stands for a smooth non-linear mapping function, and *J*(*θ*) = ∂*f* (*θ*)/∂*θ* ∈ *R^m^*^×^*^n^*.

To realize the motion tacking of this five-link planar manipulator, the inverse kinematic equation has been solved. Especially, equation (27) can be seen as a system of linear equations when the end-effector motion tracking task is allocated [i.e., $\dot{r}(t)$ is known and $\dot{\theta }(t)$ needs to be solved]. Thus, we can use the proposed FTRNN model (12) to solve this system of linear equations. Then, based on the design process of FTRNN model (12), we can obtain the following dynamic model to track control of the five-link planar manipulator [based on the formulation of equation (27)]:

$$C\dot{x}\left(t\right)=-\gamma \left(\rho_{1}\left(\mathrm{Cx}\left(t\right)-e\right)+\rho_{2}{\left(\mathrm{Cx}\left(t\right)-e\right)}^{j∕f}\right),$$

where *C* = *J*, *x* = $\dot{\theta }$ and e = $\dot{r}(t)$.

**Figure 5:**
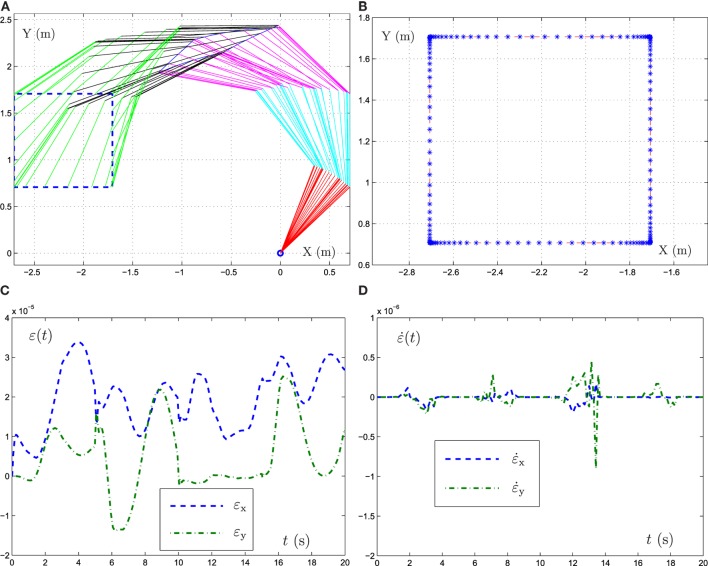
Simulative results synthesized by FTRNN model (12) when the end-effector of five-link planar manipulator tracking the square path. **(A)** Motion trajectories of manipulator, **(B)** actual and desired path, **(C)** position error, **(D)** velocity error.

**Figure 6:**
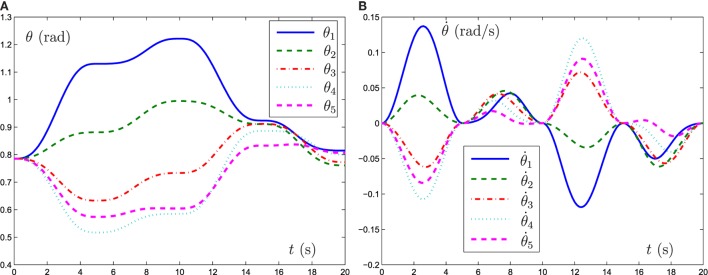
Motion trajectories of joint angle and joint velocity synthesized by FTRNN model (12) when the end-effector of five-link planar manipulator tracking the square path. **(A)** Motion trajectories of *θ*, **(B)** motion trajectories of $\dot{\theta }$.

In the simulation experiment, a square path (with the radius being 1 m) is allocated for the five-link planar manipulator to track. Besides, initial state of the mobile manipulator is set as *θ*(0) = [*π*/4, *π*/4, *π*/4, *π*/4, *π*/4]^T^, γ = 10^3^ and task duration is 20 s. The experiment results are shown in Figures 5 and 6. From the results shown in such two figures, we can obtain that the five-link planar manipulator completes the square path tracking task successfully.

## 5 Conclusion

In this paper, a finite-time recurrent neural network (FTRNN) for the complex-valued system of linear equation in complex domain is proposed and investigated. This is the first time to propose such a neural network model, which can convergence within finite time to online deal with the complex-valued system of linear equation in complex domain, and the first time to apply this FTRNN model for robotic path tracking by solving the system of linear equation. The simulation experiments show that the proposed FTRNN model has better effectiveness, as compared to the GNN model and the ZNN model for the complex-valued system of linear equation in complex domain.

## Author Contributions

LD: experiment preparation, publication writing; LX: experiment preparation, data processing, publication writing; BL: technology support, data acquisition, publication review; RL: supervision of data processing, publication review; HP revised the manuscript.

## Conflict of Interest Statement

The authors declare that the research was conducted in the absence of any commercial or financial relationships that could be construed as a potential conflict of interest. The reviewer, YZ, and handling editor declared their shared affiliation.

## References

[B1] Duran-Diaz I. Cruces S. Sarmiento-Vega M. A. Aguilera-Bonet P. (2011). Cyclic maximization of non-gaussianity for blind signal extraction of complex-valued sources. Neurocomputing 74, 2867–2873. 10.1016/j.neucom.2011.03.031

[B2] Guo D. Nie Z. Yan L. (2017). The application of noise-tolerant ZD design formula to robots’ kinematic control via time-varying nonlinear equations solving. IEEE Trans. Syst. Man Cybern. Syst. 10.1109/TSMC.2017.2705160

[B3] Guo D. Yi C. Zhang Y. (2011). Zhang neural network versus gradient-based neural network for time-varying linear matrix equation solving. Neurocomputing 74, 3708–3712. 10.1016/j.neucom.2011.05.021

[B4] He W. Chen Y. Yin Z. (2016). Adaptive neural network control of an uncertain robot with full-state constraints. IEEE Trans. Cybern. 46, 620–629. 10.1109/TCYB.2015.2411285 25850098

[B5] Hezari D. Salkuyeh D. K. Edalatpour V. (2016). A new iterative method for solving a class of complex symmetric system of linear equations. Numer. Algorithms 73, 1–29. 10.1007/s11075-016-0123-x

[B6] Jin L. Li S. (2016). Distributed task allocation of multiple robots: a control perspective. IEEE Trans. Syst. Man Cybern. Syst. 10.1109/TSMC.2016.2627579

[B7] Jin L. Li S. Xiao L. Lu R. Liao B. (2017). Cooperative motion generation in a distributed network of redundant robot manipulators with noises. IEEE Trans. Syst. Man Cybern. Syst. 10.1109/TSMC.2017.2693400

[B8] Khan M. Li S. Wang Q. Shao Z. (2016a). Formation control and tracking for co-operative robots with non-holonomic constraints. J. Intell. Robot. Syst. 82, 163–174. 10.1007/s10846-015-0287-y

[B9] Khan M. Li S. Wang Q. Shao Z. (2016b). CPS oriented control design for networked surveillance robots with multiple physical constraints. IEEE Trans. Comput. Aided Des. Integr. Circuit Syst. 35, 778–791. 10.1109/TCAD.2016.2524653

[B10] Li S. Chen S. Liu B. (2013). Accelerating a recurrent neural network to finite-time convergence for solving time-varying Sylvester equation by using a sign-bi-power activation function. Neural Process. Lett. 37, 189–205. 10.1007/s11063-012-9241-1

[B11] Li S. Li Y. (2014). Nonlinearly activated neural network for solving time-varying complex Sylvester equation. IEEE Trans. Cybern. 44, 1397–1407. 10.1109/TCYB.2013.2285166 24184789

[B12] Marco M. Forti M. Grazzini M. (2006). Robustness of convergence in finite time for linear programming neural networks. Int. J. Circuit Theory Appl. 34, 307–316. 10.1002/cta.352

[B13] Subramanian K. Savitha R. Suresh S. (2014). A complex-valued neuro-fuzzy inference system and its learning mechanism. Neurocomputing 123, 110–120. 10.1016/j.neucom.2013.06.009

[B14] Woodford G. W. Pretorius C. J. Plessis M. C. D. (2016). Concurrent controller and simulator neural network development for a differentially-steered robot in evolutionary robotics. Rob. Auton. Syst. 76, 80–92. 10.1016/j.robot.2015.10.011

[B15] Xiao L. (2015). A finite-time convergent neural dynamics for online solution of time-varying linear complex matrix equation. Neurocomputing 167, 254–259. 10.1016/j.neucom.2015.04.070

[B16] Xiao L. (2016). A new design formula exploited for accelerating Zhang neural network and its application to time-varying matrix inversion. Theor. Comput. Sci. 647, 50–58. 10.1016/j.tcs.2016.07.024

[B17] Xiao L. (2017). Accelerating a recurrent neural network to finite-time convergence using a new design formula and its application to time-varying matrix square root. J. Franklin Inst. 354, 5667–5677. 10.1016/j.jfranklin.2017.06.012

[B18] Xiao L. Liao B. Zeng Q. Ding L. Lu R. (2017a). “A complex gradient neural dynamics for fast complex matrix inversion,” in International Symposium on Neural Networks (Springer), 521–528.

[B19] Xiao L. Liao B. Jin J. Lu R. Yang X. Ding L. (2017b). A finite-time convergent dynamic system for solving online simultaneous linear equations. Int. J. Comput. Math. 94, 1778–1786. 10.1080/00207160.2016.1247436

[B20] Xiao L. Liao B. Li S. Zhang Z. Ding L. Jin L. (2017c). Design and analysis of FTZNN applied to real-time solution of nonstationary Lyapunov equation and tracking control of wheeled mobile manipulator. IEEE Trans. Ind. Inf. 10.1109/TII.2017.2717020

[B21] Xiao L. Meng W. W. Lu R. B. Yang X. Liao B. Ding L. (2015). “A fully complex-valued neural network for rapid solution of complex-valued systems of linear equations,” in International Symposium on Neural Networks 2015, Lecture Notes in Computer Science, Vol. 9377, 444–451.

[B22] Xiao L. Zhang Y. (2013). Acceleration-level repetitive motion planning and its experimental verification on a six-link planar robot manipulator. IEEE Trans. Control Syst. Technol. 21, 906–914. 10.1109/TCST.2012.2190142

[B23] Xiao L. Zhang Y. (2014a). Solving time-varying inverse kinematics problem of wheeled mobile manipulators using Zhang neural network with exponential convergence. Nonlinear Dyn. 76, 1543–1559. 10.1007/s11071-013-1227-7

[B24] Xiao L. Zhang Y. (2014b). A new performance index for the repetitive motion of mobile manipulators. IEEE Trans. Cybern. 44, 280–292. 10.1109/TCYB.2013.2253461 23757549

[B25] Xiao L. Zhang Y. (2016). Dynamic design, numerical solution and effective verification of acceleration-level obstacle-avoidance scheme for robot manipulators. Int. J. Syst. Sci. 47, 932–945. 10.1080/00207721.2014.909971

[B26] Zanchettin A. M. Ceriani N. M. Rocco P. Ding H. Matthias B. (2016). Safety in human-robot collaborative manufacturing environments: metrics and control. IEEE Trans. Autom. Sci. Eng. 13, 882–893. 10.1109/TASE.2015.2412256

[B27] Zhang Y. Ge S. S. (2005). Design and analysis of a general recurrent neural network model for time-varying matrix inversion. IEEE Trans. Neural Netw. 16, 1447–1490. 10.1109/TNN.2005.857946 16342489

[B28] Zhang Y. Shi Y. Chen K. Wang C. (2009). Global exponential convergence and stability of gradient-based neural network for online matrix inversion. Appl. Math. Comput. 215, 1301–1306. 10.1016/j.amc.2009.06.048

[B29] Zhang Y. Xiao L. Xiao Z. Mao M. (2016). Zeroing Dynamics, Gradient Dynamics, and Newton Iterations. Boca Raton: CRC Press.

[B30] Zhang Y. Yang Y. Tan N. Cai B. (2011). Zhang neural network solving for time-varying full-rank matrix Moore-Penrose inverse. Computing 92, 97–121. 10.1007/s00607-010-0133-9

